# Did the COVID-19 pandemic impact the dietary intake of individuals living with and beyond breast, prostate, and colorectal cancer and who were most likely to experience change?

**DOI:** 10.1007/s00520-023-08032-7

**Published:** 2023-09-20

**Authors:** Katie S. Taylor, Rebecca J. Beeken, Abi Fisher, Phillippa Lally

**Affiliations:** 1 Department of Epidemiology and Public Health, University College London, London, WC1E 7HB UK; 2 Leeds Institute of Health Sciences, University of Leeds, Leeds, LS2 9JT UK; 3 Department of Behavioural Science and Health, University College London, London, WC1E 7HB UK; 4 Department of Psychology, University of Surrey, Guildford, GU2 7XH UK

**Keywords:** Diet, Cancer, COVID-19, Alcohol, Snacking

## Abstract

**Purpose:**

The present work investigated dietary changes amongst individuals living with and beyond cancer (LWBC) from before to during the pandemic. To identify those at greatest risk of unhealthy changes, it was further examined whether patterns varied by sociodemographic, health-related, and COVID-19-related characteristics.

**Methods:**

This longitudinal cohort study analysed data from 716 individuals LWBC participating in the Advancing Survivorship Cancer Outcomes Trial (ASCOT). Using data provided before and during the pandemic, changes in fruit and vegetable, snack, and alcohol intake were tested using mixed-effect regression models.

**Results:**

Fruit and vegetable (95%CI: − 0.30; − 0.04) and alcohol consumption (95%CI: − 1.25; − 0.31) decreased, whilst snacking increased (95%CI: 0.19; 0.53). Women and individuals with limited social contact were more likely to reduce fruit and vegetable intake during the pandemic. Women and individuals with poorer sleep quality, limited social contact, and shielding requirements and without higher education were more likely to increase snacking during the pandemic. Individuals with poorer sleep quality, poorer mental health, and regular social contact were more likely to decrease alcohol consumption during the pandemic.

**Conclusions:**

Findings suggest decreased intake for fruit, vegetable, and alcohol consumption and increased snack intake in response to the pandemic amongst individuals LWBC. These changes appear to differ across various characteristics, suggesting the pandemic has not equally impacted everyone in this population. Findings highlight the need for targeted post-COVID strategies to support individuals LWBC most adversely affected by the pandemic, including women and socially isolated individuals. This encourages resources to be prioritised amongst these groups to prevent further negative impact of the pandemic. Whilst the findings are statistically significant, practically they appear less important. This is necessary to acknowledge when considering interventions and next steps.

**Supplementary Information:**

The online version contains supplementary material available at 10.1007/s00520-023-08032-7.

## Introduction

The COVID-19 pandemic has negatively impacted people’s lives, including their physical and mental health, financial security, and social relationships [1]. Research also indicates an impact of the pandemic on health behaviours [2], including diet [3]. Changes in diet may be due to increased stress resulting in emotional eating [4], restricted access to fresh foods [5], changing working environments [6], and fear of the virus [7]. Since the pandemic started, studies exploring dietary changes suggest the consumption of fruits and vegetables [8–12], snacks [8, 10–13], and alcohol [8, 11, 13] has both increased and decreased. These mixed findings suggest a need to investigate factors associated with the differences in dietary change, as certain groups of individuals may have changed their behaviours in different ways [14].

Identifying factors associated with dietary change generally, and during the pandemic, is important for identifying those at greatest risk. Several sociodemographic variables associated with diet across the life course have been identified. Diet quality appears to follow a socioeconomic gradient; one study observed that higher baseline education levels predicted increased healthier dietary patterns over 4 years [15]. This observed gradient may be due to lack of nutritional education [16] or financial resources [17]. Contrastingly, during the COVID-19 lockdown, research observed that those with a high education level ate less healthily and purchased more snacks compared to those with low education levels [18]. This may be due to individuals with higher education being more likely to work from home during lockdown which likely influenced eating behaviours [19]. Research has also established differences between males and females regarding their health behaviours and motivations for adhering to positive health behaviours [20]. For example, females generally place greater importance on healthy eating than males [21]. During the COVID-19 pandemic, one study using data from 5 British cohort studies observed that men had higher alcohol consumption than women and reported lower fruit and vegetable intake both before and during the pandemic [22].

Health-related factors also influence dietary intake. Short sleep duration and poor sleep quality are associated with increased snacking and preference for energy-dense foods [23]. Mental health conditions have also been associated with poorer diet quality. One study of 1634 adults from the Netherlands observed diet quality to be worse amongst participants with a current anxiety or depressive disorder than amongst healthy controls [24]. Research conducted during the pandemic identified an association between higher stress and unhealthy eating practices [25]. These health factors are important as sleep [26] and mental health [27] have been impacted by the pandemic.

The pandemic has led to increased social isolation due to imposed social restrictions. Individuals at greater risk from the virus due to existing health conditions were advised to ‘shield’, which furthered isolation. Evidence suggests that social factors such as frequency of social contact and social engagement are important influences on diet [28, 29]. Socially isolated older adults appear to be especially vulnerable to dietary inadequacy, including minimal consumption of fruit and vegetables, due to a lack of social support [30]. Considering the increase in social isolation during the pandemic [31], it is critical to understand how this change has impacted diet.

A key population for whom health behaviours are particularly important are individuals living with and beyond cancer (LWBC). Currently, 2.5 million people are estimated to be LWBC in the UK, rising to 4 million by 2030 [32]. Cancer survivorship benefits from healthy lifestyle factors; adopting the healthiest lifestyle is associated with a 52% lower cancer mortality risk compared with having the least healthy lifestyle [33]. The World Cancer Research Fund (WCRF) has provided a set of dietary recommendations for those LWBC including to (a) eat at least 30g of fibre and 400g of fruit and vegetables every day, (b) limit fast foods and other processed foods high in fat, starches, or sugars, (c) limit red meat consumption to 500g per week and avoid processed meat, and (d) not drink alcohol. Research has suggested that dietary behaviours may improve following a cancer diagnosis, including increased fruit and vegetable consumption and reduced intake of sugar and sweets [34–36]. A recent systematic review observed that a better overall diet may improve survival and quality of life after a breast cancer diagnosis and a Mediterranean diet may be protective for overall mortality following a colorectal cancer diagnosis [37]. Inflammation may act as a potential pathway for these associations as higher quality diets post-diagnosis were associated with lower C-reactive protein levels in cancer patients [38] and both a Mediterranean diet and lower Healthy Eating Index diet scores are considered to have low inflammatory potential [39]. This demonstrates the beneficial effects of a healthy dietary pattern on cancer survival in terms of mortality but also for quality of life. However, the dietary impact of the pandemic amongst individuals LWBC and the individuals LWBC most vulnerable to unhealthy dietary changes is unknown. It is important to identify those most likely to experience dietary change during the pandemic as this can inform the development of post-COVID targeted strategies to support those most adversely affected by the pandemic.

Using longitudinal data from the Advancing Survivorship Cancer Outcomes Trial (ASCOT), we evaluated whether diet (fruit and vegetable, snack, and alcohol consumption) was different during the COVID-19 pandemic compared with before the pandemic. We also investigated whether dietary patterns varied with sociodemographic (gender, education), health-related (sleep quality, anxiety, and depression), and COVID-19-related factors (shielding, social contact). Two research questions were constructed: (1) Has the intake of fruit and vegetables, snacks, and alcohol changed for individuals LWBC from before to during the pandemic? (2) What sociodemographic, health-related, and COVID-19-related predictors influenced dietary change amongst individuals LWBC during the pandemic?

## Methods and materials

### Design and procedure

This study is part of ASCOT, a 2-arm randomised controlled trial that tested a brief habit-based health behaviour intervention for individuals LWBC [40]. The trial included 1348 patients diagnosed with non-metastatic (stages I–III) breast, prostate, or colorectal cancer, recruited from seven NHS trusts across London and Essex. Surveys were completed on paper, online, or via telephone with a researcher at baseline, 3 months, 6 months, and 2 years post-recruitment with the addition of a COVID-19 follow-up. The current research used the participants from ASCOT as a cohort rather than assessing intervention effectiveness. The sample included participants who, before the pandemic began, had completed all main trial assessments (3 months, 6 months, and 2 years) and those who had completed the 6-month assessment but were not yet due for the 2-year assessment. Questionnaire data was used from two timepoints: participant’s most recent assessment before the pandemic and during the pandemic. The most recent questionnaires completed before the pandemic were collected between April 2018 and March 2020, and the COVID-19 follow-up was completed between September 2020 and May 2021.

### Sample

The participants were adult non-metastatic breast, prostate, or colorectal cancer patients who received their diagnosis between 2006 and 2016, had completed primary curative treatment, and expressed interest in participating in a lifestyle programme. Participants were excluded at baseline if they were receiving active treatment for cancer or were not able to consent due to severe cognitive impairment. Included in the current analyses were participants with valid data for fruit and vegetable, snack, and alcohol consumption at both the before pandemic and during pandemic assessment points (*N* = 716).

### Measures

#### Outcome variables

Fruit and vegetable consumption was measured using a 2-item measure requiring participants to separately state how many portions of fruit and vegetables they usually ate over the past month, with possible responses ranging from 1 (‘less than one per week’) to 7 (‘3 or more per day’). These two responses were converted into a number that reflected the number of portions per day and then summed to create a combined daily fruit and vegetable score (*range* 0–7) [41] which has been validated against biomarkers [42].

Snack food consumption was measured using an item from the Dietary Instrument for Nutrition Education (DINE) [43]. Participants stated how many times a week they ate a serving of biscuits, chocolate, or savoury snacks (e.g. crisps, nuts). Possible responses ranged from 1 (‘less than once a week’) to 4 (‘6 or more per week’). Answers were converted into a number that reflected the number of portions per week.

Finally, alcohol consumption was measured using 2 items adapted from the AUDIT-C. Firstly, how often do you have a drink containing alcohol with the responses of ‘never’, ‘monthly or less’, ‘2–4 times per month’, ‘2–3 times per week’, ‘4–5 times per week’, or ‘everyday’. Secondly, how many units of alcohol do you drink on a typical day when you are drinking with the responses ‘I never drink alcohol’, ‘1–2’, ‘2–4’, ‘5–6’, ‘7–9’, or ‘10 + ’ units. These two responses were then converted to numbers and multiplied to create weekly units of alcohol, ranging from 0 to 70 units per week.

#### Exposure variables

Several exposure variables were assessed, including sociodemographic, health-related, and COVID-19-related variables. The sociodemographic variables included were gender (male (reference category) versus female) and education (higher education (reference category) versus no higher education).

The health-related variables included were mental health and sleep quality during the pandemic. Mental health was assessed using a single item from the EQ-5D developed by the Euroqol Group [44]. The validity and reliability of this measure amongst cancer patients have been supported [45]. Participants selected 1 of 5 responses that best describes their health today. For anxiety and depression, responses ranged from ‘I am not anxious or depressed’ to ‘I am extremely anxious or depressed’. Due to a small number of participants in certain groups, responses were categorised into ‘no symptoms’ (reference category), ‘minimal symptoms’, and ‘high symptoms’. It has been suggested that a single question on depressed mood can detect 85–90% of patients with major depressive disorder [46]. Sleep quality was assessed using the Pittsburgh Sleep Quality Index (PSQI), a 19-item self-report questionnaire that assesses sleep quality and quantity and ranges from 0 to 21 (higher scores indicating worse sleep quality). The questionnaire yields 7 component scores: subjective sleep quality, sleep latency, duration, habitual sleep efficiency, sleep disturbances, use of sleep medication, and daytime dysfunction. Items 5d and 5j and 10a–e of the PSQI were omitted for the present survey and scoring was adjusted accordingly. Psychometric evaluation supports its internal consistency, reliability, and construct validity amongst cancer patients [47].

The COVID-19-related variables included were the requirement to shield and social contact during the pandemic. For shielding, participants were asked whether they received a letter from the government stating they were required to shield as they were at high risk from the virus. Responses were ‘yes’ or ‘no’ (reference category). Social contact during the pandemic was assessed by asking participants the activities they were completing outside of their house at the time they completed the questionnaire. Possible responses included ‘not leave home at all’, ‘only leave for exercise or medical appointments’, ‘only leave for exercise, medical appointments, food shopping or to collect medication’, ‘only leave for exercise, medical appointments, food shopping, collect medication, or work’, ‘not restrict where you went but socially distanced when near others’, or ‘not restrict where you went or how far away you were from others’. Due to a small number of participants in certain groups, these were categorised into limited (responses 1–3) or regular social contact (4–6) (reference category).

## Statistical analyses

### Main analyses

Statistical analyses were conducted in R and separate analyses were conducted for each of the dietary components. Descriptive statistics are reported as means (*M*) and standard deviations (SD) or *n* (%). Missing data for all included exposure and outcome variables was below 5%. Linear mixed-effect models were conducted using the *lme4* package to estimate changes in fruit and vegetable, snack, and alcohol intake during the COVID-19 pandemic. To address the first research question, changes in the dietary outcomes were estimated using a binary predictor variable that indicated whether the outcome was measured before or during the pandemic, which we shall name the time indicator. To address the second research question, interaction effects between the time indicator and the sociodemographic, health-related, and COVID-19-related variables were examined in separate models to understand whether and how the change in dietary outcomes might vary across different characteristics. The covariates that were included in the analyses were based on previous research and included age, gender, ethnicity, and education.

### Sensitivity analyses

Subgroup analyses were conducted to understand whether the participants excluded from the current sample, due to the diet-related exclusion criteria, differed meaningfully from those included.

## Results

A total of 716 participants out of 1348 at baseline (53.1%) were included in the current analyses. Participant characteristics are presented in Table 1. Participants had a mean age of 66 years at the COVID-19 follow-up, 62.3% were female, 94.1% were of white ethnicity, and 37.7% had higher education. Regarding mental health, 54.6%, 33%, and 12.4% of participants reported feeling no, low, and strong feelings of anxiety and/or depression. Average sleep quality score was ~ 6.82 (± 3.49; *range* = 0–19). For COVID-19-related factors, only 17.7% of participants were sent a letter saying they needed to shield and 49.9% had limited social contact during the pandemic. Average intake for each of the dietary outcomes is presented in Table 2. Before the pandemic, average daily portions of fruits and vegetables were ~ 4.20 (± 2.07; *range* = 0–7) compared to ~ 4.02 (± 2.01; *range* = 0–7) during the pandemic. Before the pandemic, average weekly portions of snacks were ~ 2.45 (± 2.13; *range* = 0–7) compared to ~ 2.78 (± 2.23; *range* = 0–7) during the pandemic. Finally, average weekly alcohol consumption before the pandemic was ~ 6.24 (± 10.11; *range* = 0–70) compared to ~ 5.52 (± 9.22; *range* = 0–70) during the pandemic.

**Table 1:** Sample characteristics

Variable	Overall (*N* = 716)
Age	
Mean (SD)	66.48 (10.68)
Range	34.09–89.20
Gender	
Male	270 (37.7%)
Female	446 (62.3%)
Ethnicity	
Missing	2 (0.3%)
White	672 (94.1%)
Other	42 (5.9%)
Education	
Missing	34 (4.7%)
Does not have higher education	425 (62.3%)
Has higher education	257 (37.7%)
Anxiety and depression	
No feelings	391 (54.6%)
Some feelings	236 (33.0%)
Strong feelings	89 (12.4%)
Sleep quality	
Missing	6
Mean (SD)	6.82 (3.49)
Range	0–19
Shielding	
Yes	589 (82.3%)
No	127 (17.7%)
Social contact	
Limited	352 (49.2%)
Regular	364 (50.8%)

**Table 2:** Descriptives for the dietary outcomes (*N* = 716)

Variable	Before the pandemic	During the pandemic
Fruit and vegetables		
Mean (SD)	4.20 (2.07)	4.02 (2.01)
Range	0–7	0–7
Snacks		
Mean (SD)	2.45 (2.13)	2.78 (2.23)
Range	0–7	0–7
Alcohol		
Mean (SD)	6.24 (10.11)	5.52 (9.22)
Range	0–70	0–70

### Mixed effect individual models

The independent mixed effect models are summarised in Table 3. Fruit and vegetable consumption decreased from before to during the pandemic by 0.17 portions per day (95%CI: − 0.30; − 0.04). Snack intake increased from before to during the pandemic by 0.36 portions per week (95%CI: 0.19; 0.53). Alcohol intake decreased from before to during the pandemic by 0.78 units (95%CI: − 1.25; − 0.31).

**Table 3:** Mixed effect main models (*N* = 716) for fruit and vegetable, snack, and alcohol intake

Predictors	Estimates (b)	CI low	CI high	*P* value	FDR
Fruit and Veg^1^					
Wave	− 0.17	− 0.3	− 0.04	0.01	0.01
Snacks^1^					
Wave	0.36	0.19	0.53	< 0.001	0.002
Alcohol^1^					
Wave	− 0.78	− 1.25	− 0.31	< 0.001	0.002

^1^Covariates included age, gender, education, and ethnicity

### Mixed effect interaction models

The mixed effect interaction models are summarised in Table 4. The interaction with gender suggests that males did not change their fruit and vegetable intake from before to during the pandemic, whereas females decreased their intake by 0.27 portions (interaction effect =  − 0.27; 95%CI: − 0.54; 0.00). Likewise, snack intake for males increased from before to during the pandemic by 0.07 portions per week compared to 0.52 portions for females (interaction effect = 0.45; 95%CI: 0.10; 0.80). Individuals with higher education increased their snack intake by 0.12 portions per week from before to during the pandemic, compared to a 0.50 increase for those without higher education (interaction effect = 0.38; 95%CI: 0.03; 0.73).

**Table 4:** Mixed effect interaction models for fruit and vegetable, snack, and alcohol intake

Predictors	Estimates	CI low	CI high	*P* value	FDR^3^
Fruit and veg^1,2^					
Wave*Gender	− 0.27	− 0.54	0.00	0.05	0.09
Wave*Education	0.06	− 0.21	0.33	0.69	0.77
Wave*Anxiety (low)	− 0.02	− 0.31	0.26	0.86	0.86
Wave*Anxiety (high)	0.04	− 0.37	0.45	0.86	0.86
Wave*Sleep	− 0.01	− 0.05	0.02	0.41	0.57
Wave*Shield	0.2	− 0.14	0.54	0.24	0.36
Wave*Social	0.28	0.02	0.54	0.04	0.07
Snacking^1,2^					
Wave*Gender	0.45	0.10	0.80	0.01	0.05
Wave*Education	0.38	0.03	0.73	0.03	0.07
Wave*Anxiety (low)	0.14	− 0.23	0.52	0.45	0.57
Wave*Anxiety (high)	0.50	− 0.04	1.03	0.07	0.12
Wave*Sleep	0.06	0.02	0.11	0.01	0.03
Wave*Shield	0.75	0.30	1.19	< 0.001	0.01
Wave*Social	0.56	0.23	0.90	< 0.001	0.01
Alcohol^1,2^					
Wave*Gender	− 0.16	− 1.13	0.82	0.75	0.83
Wave*Education	0.82	− 0.15	1.78	0.10	0.16
Wave*Anxiety (low)	− 1.11	− 2.14	− 0.08	0.03	0.07
Wave*Anxiety (high)	− 1.68	− 3.16	− 0.21	0.03	0.07
Wave*Sleep	− 0.13	− 0.26	− 0.01	0.03	0.07
Wave*Shield	0.46	− 0.77	1.70	0.46	0.57
Wave*Social	1.27	0.34	2.21	0.01	0.04

^1^Covariates included age, gender, education, and ethnicity

^2^The reference categories were being male, having higher education, no feelings of anxiety or depression, not shielding, and regular social contact

^3^*FDR*, false discovery rate; results after adjustment for multiple testing

Participants with high (~ 1.48 units) (interaction effect =  − 1.68; 95%CI: − 3.16; − 0.21) and low (~ 0.91 units) (interaction effect =  − 1.11; 95%CI: − 2.14; − 0.08) feelings of anxiety or depression showed greater decreases in alcohol consumption compared to those with no feelings of anxiety or depression (~ 0.2 units). Individuals with poorer sleep quality had a greater increase in snacking compared to those with better sleep quality (interaction effect = 0.06; 95%CI: 0.02; 0.11). For example, participants with a sleep score of 1, indicating good quality sleep, did not change their weekly snack intake from before to during the pandemic, compared to individuals with a sleep score of 19, indicating poor quality sleep, increased their snacking intake by 1.08 portions per week. Individuals with poorer quality sleep have a greater decrease in alcohol consumption compared to those with better quality sleep (interaction effect =  − 0.13; 95%CI: − 0.26; − 0.01). For example, participants with a sleep score of 1, indicating good sleep quality, decreased their alcohol consumption by 0.01 units compared to a decrease of 2.35 units for those with a score of 19, indicating poor sleep quality.

Participants who received a letter recommending them to shield showed a greater increase in snacking (~ 0.98 portions per week) compared to those who did not (~ 0.23 portions per week) (interaction effect = 0.75; 95%CI: 0.30; 1.19). Participants with regular social contact showed a greater decrease in fruit and vegetable intake (~ 0.31 portions) compared to those with limited social contact (~ 0.03 portions) (interaction effect = 0.28; 95%CI: 0.02; 0.54). Participants with regular social contact increased their snack intake by 0.09 portions per week from before to during the pandemic, compared to 0.56 portions for individuals with limited social contact (interaction effect = 0.56; 95%CI: 0.23; 0.90). Participants with regular social contact decreased their alcohol consumption by 1.39 units compared to a decrease of 0.12 units for those with limited social contact (interaction effect = 1.27; 95%CI: 0.34; 2.21).

There were no changes in fruit and vegetable consumption from before to during the pandemic by education, mental health, sleep, or shielding. Likewise, mental health was not associated with changes in snacking and gender, education, and shielding were not associated with changes in alcohol consumption.

## Sensitivity analyses

The subgroup analyses (Supplementary Table 1) comparing participants that were excluded (*N* = 632) and included (*N* = 716) in the current sample suggest that the samples differed by age, ethnicity, education level, and number of comorbidities but not by gender or index of multiple deprivation.

## Discussion

The current research observed dietary changes amongst individuals LWBC during the COVID-19 pandemic. Specifically, fruit, vegetable, and alcohol consumption decreased whilst snacking increased. Changes in dietary habits were influenced by sociodemographic, health-related, and COVID-19-related factors. Women and individuals with limited social contact were more likely to reduce fruit and vegetable intake during the pandemic. Women and individuals with poorer sleep quality, limited social contact, and shielding requirements and without higher education were more likely to increase snacking during the pandemic. Individuals with poorer sleep quality, greater anxiety and depression levels, and regular social contact were more likely to decrease alcohol consumption during the pandemic.

The first research question aimed to identify changes in the consumption of fruit and vegetables, snacking, and alcohol amongst individuals LWBC from before to during the pandemic. Within this sample, fruit and vegetable consumption appeared to decrease from before to during the pandemic which is supported by existing research [48]. Possible explanations for this finding include disruption to the food supply chain during the pandemic [49] impacting food availability [50, 51], leading to shifting preferences towards non-perishable foods over fresh foods, including fruit and vegetables [49]. Likewise, the negative economic impact of the pandemic [52, 53] may have encouraged a reduction in purchasing fruit and vegetables as they are perceived as more expensive [54]. Furthermore, increased stress and anxiety levels amongst individuals LWBC [55] may have led to increased snacking and reduced fruit and vegetable consumption [48, 56, 57]. Previous research identifies an association between ‘natural disasters’ and reduced fresh produce consumption [58], explained via a combination of higher anxiety levels, increased food prices, and decreased availability, which could be likened to the pandemic.

Research supports the finding that snacking increased during the pandemic for individuals LWBC. During the pandemic, increased snacking has been observed alongside elevated stress, boredom, and emotional eating [59–61]. Given the high palatability of snacks, they may have acted as a comfort mechanism to deal with COVID-19-related stress [60, 62]. Foods high in saturated fat, salt, and sugar (HFSS) also tend to be cheaper and have long shelf lives. Individuals may have ‘stocked-up’ on HFSS foods through less frequent shopping trips [60]. Home confinement appears to have increased sedentary time amongst breast cancer patients during the pandemic up to 54.2% [63], which may have further contributed to increased snacking [64].

The finding that alcohol consumption decreased amongst individuals LWBC during the pandemic is less expected. A systematic review of general and clinical populations observed an overall trend towards increased alcohol consumption [65]. However, alcohol consumption may have decreased due to reduced socialising following restrictions imposed by the government, and the current sample may have been comprised of primarily social drinkers. Furthermore, research suggests that following a cancer diagnosis individuals LWBC reduce overall consumption of alcohol to improve survival chances and reduce likelihood of re-diagnosis [66]. This protective behaviour may therefore have been established before the pandemic which could have limited any negative changes during the pandemic.

The second research question aimed to explore the sociodemographic, health-related, and COVID-19-related factors associated with dietary changes during the pandemic. The current findings observed that females, compared to males, consumed more snacks and less fruit and vegetables during the pandemic compared to before. Women have a higher tendency to increase their snacking in response to negative affect [67, 68] which is suggested to have increased during the pandemic [69]. Therefore, females may be more responsive to COVID-related stressors, resulting in increased snacking. Research showed that men were more likely to adhere to a Mediterranean diet, including intake of fruit and vegetables, during the pandemic compared to women which supports the current findings.

Individuals with no higher education, compared to those with higher education, were found to increase snack consumption during the pandemic. Human capital theory indicates that education increases income [70], suggesting individuals with lower education levels likely have lower income. Considering the financial impact of the pandemic, less socioeconomically advantaged individuals may be more likely to prioritise foods with longer shelf lives including snacks [49]. Likewise, these disadvantaged individuals may be more likely to experience COVID-19-related stress due to financial insecurity [71] which may encourage emotional eating and a preference for HFSS foods [72].

Health-related and COVID-19-related variables were also associated with increased snacking amongst individuals LWBC during the pandemic. Shielding and limited social contact were associated with increased snacking during the pandemic, compared to before. These individuals are likely experiencing higher levels of social isolation compared to others [73]. Various periods of isolation can have long-term effects on health, including psychiatric symptoms [74]. Psychiatric symptoms have been associated with emotional eating which contributes to snack intake [75]. Individuals with worse sleep also consumed more snacks during the pandemic which is supported by a plethora of research highlighting that insufficient sleep increases snacking [23].

Reduced alcohol consumption was predicted by various health-related and COVID-19-related factors. Individuals with poorer mental health consumed less alcohol during the pandemic compared to before. Research usually observes the opposite, with poorer mental health predicting increases in alcohol consumption [76]. Perhaps individuals with poorer mental health may be less likely to be drinking socially compared to those with better mental health during the pandemic. More research is necessary to understand this association. Individuals with poorer sleep quality consumed less alcohol during the pandemic. Research commonly finds an association between worse sleep quality and increased alcohol consumption [77]. However, these individuals may have tried to rectify their poor sleep during the pandemic by reducing their alcohol intake. This potential explanation warrants further longitudinal exploration. Finally, individuals with regular social contact consumed less alcohol during the pandemic. More frequent social contact during the pandemic was associated with lower anxiety [78] which may lead to reduced alcohol consumption. Therefore, stress-related mental health may be a mediator between social contact and alcohol consumption.

To our knowledge, this is the first paper to prospectively investigate the dietary changes amongst individuals LWBC during the COVID-19 pandemic. A strength of the current study is the larger sample size compared to previous similar research which provides greater power to detect differences. The longitudinal design also allows the tracking of behaviour change over time, allowing behavioural patterns and determinants to be identified.

Nonetheless, the findings should be viewed in the context of several limitations. The sample may not be representative of the target population as some subgroups may have been unequally represented. The subgroup analyses comparing participants that were excluded and included in the sample demonstrated differences in age, ethnicity, and education. These differences increase the likelihood of selection bias and question the generalisability of the findings. Considering the outcome measures were self-reported and the sensitive nature of several measurements collected, including mental health, the research may be subject to self-report bias or social desirability bias. Furthermore, as we included only one pre-pandemic timepoint, we cannot determine whether the effects observed were a response to the pandemic or due to a pre-existing behavioural trend. Finally, whilst the changes in dietary intake are statistically significant, this may not translate into practical or clinical significance considering the portion changes are small.

The current results have potential implications for both individuals LWBC and the general population by providing a more detailed understanding of the potential risk factors for dietary change during the pandemic. Specifically, the findings may suggest a need for more targeted interventions post-COVID based on the risk factors identified. This could allow resources to be prioritised amongst these groups to prevent or reduce any further negative impact of the pandemic. In particular, considering the impact of the pandemic on social contact and the suggested greater risk of less healthy dietary changes for the more socially isolated individuals, interventions that promote social support and social contact amongst those LWBC may be important, for example encouraging in-person or online group support sessions for individuals LWBC that perhaps focus on dietary behaviour change.

To conclude, this study suggests that individuals living with and beyond cancer may have decreased their fruit, vegetable, and alcohol intake and increased their snack intake in response to COVID-19. These changes appear to be different based on sociodemographic, health-related, and COVID-19-related factors, which suggests that the pandemic has not equally impacted everyone. Future research should focus on understanding the potential mediators explaining the observed associations to better understand the pathways to unhealthy dietary change.

### Supplementary Information

Below is the link to the electronic supplementary material.

Supplementary file1 (DOCX 21 KB)

## Data Availability

The datasets generated during and/or analyzed during the current study are available from the corresponding author on reasonable request.
