# Supplementary material for: Did the COVID-19 pandemic impact the dietary intake of individuals living with and beyond breast, prostate, and colorectal cancer and who were most likely to experience change?
Source: Support Care Cancer. 2023 Sep 20;31(10):585. doi: 10.1007/s00520-023-08032-7 (PMC10511549; doi:10.1007/s00520-023-08032-7)
Supplement: Supplementary file 1 — Supplementary file1 (DOCX 21 KB) [file 520_2023_8032_MOESM1_ESM.docx]

**Supplementary material**

**Article title:** Did the COVID-19 pandemic impact the dietary intake of individuals living with and beyond breast, prostate, and colorectal cancer and who was most likely to experience change?

**Journal:** Supportive Care in Cancer

**Authors:** Katie S Taylor, MSc^1^, Rebecca J Beeken, PhD^2^, Abi Fisher, PhD^3^, Phillippa Lally, PhD^4^

^1^ Department of Epidemiology and Public Health, University College London, WC1E 7HB, London, UK.

^2^ Leeds Institute of Health Sciences, University of Leeds, Leeds, LS2 9JT, UK.

^3^ Department of Behavioural Science and Health, University College London, WC1E 7HB, London, UK.

^4^ Department of Psychology, University of Surrey, GU2 7XH, Guildford.

Correspondence to: Rebecca Beeken ([r.beeken@leeds.ac.uk](mailto:r.beeken@leeds.ac.uk)).

Supplementary Table 1: Comparison of the characteristics of the analytical sample versus excluded participants at baseline.

|  | **Complete (N=716)** | **Incomplete (N=632)** | **P value** |
| --- | --- | --- | --- |
| **Age** |  |  |  |
| Mean (SD) | 62.84 (10.75) | 65.82 (11.94) | 0.02 |
| **Sex** |  |  |  |
| Male | 270 (37.7%) | 250 (39.6%) | 0.49 |
| Female | 446 (62.3%) | 382 (60.4%) |  |
| **Ethnicity** |  |  |  |
| White | 672 (94.1%) | 570 (90.8%) | 0.01 |
| Ethnic minority | 42 (5.9%) | 58 (9.2%) |  |
| **Education** |  |  |  |
| None | 91 (13.3%) | 135 (23.6%) | <0.001 |
| GCSEs | 235 (34.5%) | 177 (30.9%) |  |
| A-levels | 99 (14.5%) | 75 (13.1%) |  |
| Degree or above | 257 (37.7%) | 185 (32.3%) |  |
| **IMD** |  |  |  |
| Mean (SD) | 6.60 (2.46) | 6.20 (2.53) | 0.48 |
| **Comorbidities** |  |  |  |
| Mean (SD) | 1.06 (1.16) | 1.35 (1.40) | <0.001 |
